# A refractory tenosynovitis of the wrist: a case report

**DOI:** 10.1186/s13256-022-03278-x

**Published:** 2022-02-21

**Authors:** Alex Boudon, Onya Opota, Diana Dan

**Affiliations:** 1 Rheumatology, University Hospital of Lausanne and Lausanne University, Avenue Pierre-Decker 4, 1005 Lausanne, Switzerland; 2 Institute of Microbiology, University Hospital of Lausanne and Lausanne University, Lausanne, Switzerland

**Keywords:** *Mycobacterium malmoense*, Nontuberculous mycobacteria, Refractory tenosynovitis, Finger flexor tenosynovitis, Case report

## Abstract

**Background:**

*Mycobacterium malmoense* is a species of slow-growing nontuberculous mycobacteria. It causes mostly pulmonary infections or lymphadenitis in children, but is increasingly encountered in isolated tenosynovitis in adults. Diagnosis is often delayed because of the rarity of the condition and the difficulty of culturing the bacteria.

**Case presentation:**

We report on a rare association of seronegative polyarthritis with infectious nontuberculous mycobacteria tenosynovitis. A 65-year-old Caucasian female was referred to our clinic because of persisting tenosynovitis of the finger flexor tendons of her right hand, despite two previous synovectomies. She also reported bilateral shoulder and left wrist pain. Paraclinical investigations showed slightly elevated inflammatory parameters. Ultrasound showed synovitis of metacarpophalangeal joints of the right hand and right knee, and a bilateral subacromial bursitis. Hand magnetic resonance imaging also revealed an erosive carpal synovitis. Bacteriological analysis of the second tenosynovectomy specimen showed no growths in aerobic and anaerobic cultures. An additional synovial fluid analysis of the wrist joint was negative for mycobacteria and crystals. Seronegative polyarthritis was suspected, but the initiated immunosuppressive treatment with prednisolone and methotrexate resulted in no clinical improvement of the tenosynovitis. Yet the other joints responded well, and the inflammatory parameters normalized. The immunosuppression was later stopped because of side effects. Due to massive worsening of the tenosynovitis, a third synovectomy was performed. *Mycobacterium malmoense* was identified on biopsy, leading to the diagnosis of infectious tenosynovitis. At this point, we started an antituberculous therapy, with incomplete response. A combination of antimicrobial and immunosuppressive treatment finally led to the desired clinical improvement.

**Conclusion:**

The treatment of nontuberculous mycobacteria tenosynovitis is not well established, but combining antibiotics with surgical debridement is probably the most adequate approach. Our case highlights the importance of having a high clinical suspicion of an atypical infection in patients with inflammatory tenosynovitis not responding to usual care.

## Background

Nontuberculous mycobacteria (NTM) are saprophytic bacteria mostly found in water and soil. Usually nonpathogenic, they can cause opportunistic infections of various localizations in immunocompromised patients, but are increasingly recognized also in immunocompetent patients. About 90% of cases involve the lungs, and the remaining 10% involve the lymph nodes, the skin, and the musculoskeletal system [1–3]. Out of more than 120 NTM species, only a few are known to cause osteoarticular infections (mainly of the hand), the most common being *Mycobacterium marinum* and the second most common being *Mycobacterium kansasii*, with far fewer infections caused by other NTM [4]. *Mycobacterium malmoense*, a slow-growing NTM species, causes pulmonary infections (in about 80% of cases) in immunocompromised or predisposed patients. The main extrapulmonary disease is cervical lymphadenitis in children. Isolated tenosynovitis is an extremely rare manifestation, with only 11 cases described in the literature [5]. Out of these 11 patients, only two had a previous inflammatory rheumatic disease and, unlike our patient, they were both on immunosuppressive therapy prior to the infection. The diagnosis of NTM tenosynovitis is often delayed, mostly because of the rarity of the condition, but also because of the difficulty of culturing the bacteria [6]. The awareness about this type of infection is even lower in case of immunocompetent individuals. Left untreated, this pathology may lead to substantial morbidity and unnecessary interventions. It is therefore paramount to recognize and treat it in a timely manner. The therapy for a concomitant rheumatic disease needing immunosuppression may be challenging, as we will discuss later on. We therefore think that our case could help increase the awareness of atypical mycobacterial infections and improve the management of patients.

## Case presentation

**Fig. 1:**
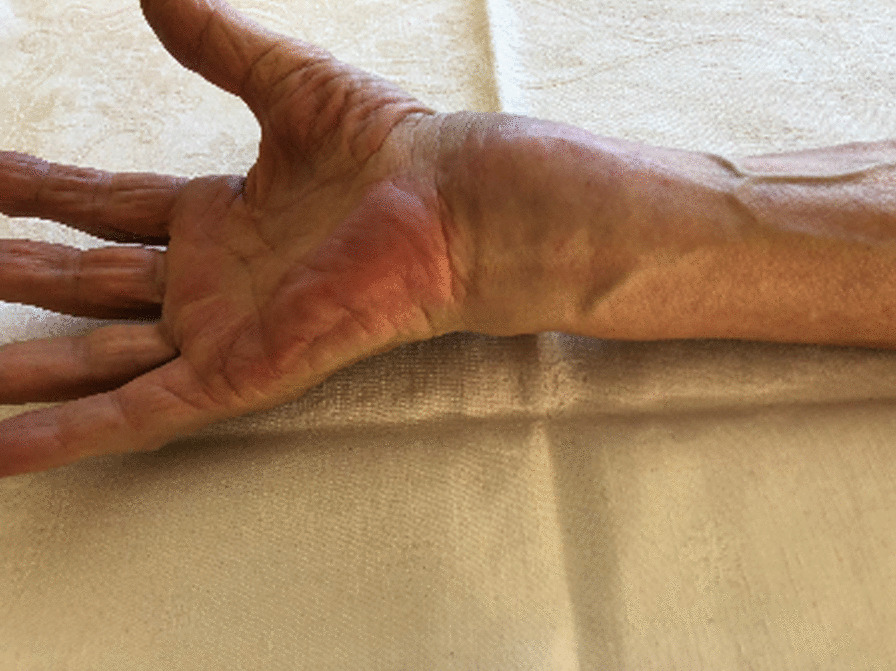
Massive swelling of the volar side of the right wrist, accompanied by amyotrophy of hand muscles

We report on a 65-year-old Caucasian female patient who presented in March 2017 with persistent right wrist pain and swelling (Fig. 1).

She suffered from arterial hypertension and asymptomatic cerebral aneurysm and had chronic alcohol (2–3 units/day) and tobacco consumption (50 pack-years). She had a history of surgery for a trigeminal neuralgia 4 years previously. She took amlodipine [10 mg/day* per os* (PO)] and aliskiren (300 mg/day PO) for her hypertension and low-dose aspirin (100 mg/day PO) for the cerebral aneurysm. She had no history of prior tuberculosis, thrombosis, or miscarriage or abortion. Her family history was negative for autoimmune, rheumatic diseases and psoriasis. She did not have an aquarium or any contact with animals and had not traveled in the previous year, but she gardened. She was a retired waitress, was married, and had a son.

The patient had no inflammatory low back pain, respiratory symptoms, psoriasis, or uveitis. She had no history of trauma, but she had a carpal tunnel release the previous year. During surgery, a tenosynovitis of all deep and superficial finger flexor tendons was detected, so a tenosynovectomy was performed. As soon as 2 months later, the tenosynovitis reoccurred and a second tenosynovectomy was performed. Still, the patient suffered a second relapse after another 4 months, and was referred to our rheumatology clinic for further investigation. Besides right wrist pain, she also complained of left wrist and bilateral shoulder pain. Blood pressure was 142/86 mmHg, pulse 64 beats per minute, body temperature 36.7 °C, height 157 cm, and body weight 50 kg. Joint examination showed no abnormalities besides the flexor-tendon tenosynovitis, and general examination was normal, including lymph node status as well as heart and lung auscultation. Equally, there was no hepatomegaly or splenomegaly. Neurologic evaluation showed normal gait, strength, and sensibility.

### Laboratory investigations

Paraclinical investigations showed slightly elevated inflammatory parameters (C-reactive protein 32 mg/L, erythrocyte sedimentation rate 28 mm per hour, leukocytes 12.8 G/L, neutrophils 11.65 G/L, lymphocytes 0.64 G/L, thrombocytes 430 G/L), normal hemoglobin (157 g/L), and no eosinophils or basophils. Antinuclear antibodies were at 1:160, without specificity, and rheumatoid factor, cyclic citrullinated peptide antibodies, and HLA B-27 were negative. The following parameters were normal: corrected calcium (2.36 mmol/L), phosphate (1.01 mmol/L), creatinine (56 μmol/L), urate (286 μmol/L), iron (17.3 μmol/L), transferrin (31 μmol/L), transferrin saturation coefficient (0.28), glutamic-oxaloacetic transaminase (GOT) (24 U/L), glutamic pyruvic transaminase (GPT) (26 U/L), alkaline phosphatase (78 U/L), gamma-glutamyl transferase (GGT) (23 U/L), 25(OH) vitamin D (28.4 μg/L), parathyroid hormone (10 ng/L), and thyroid-stimulating hormone (1.27 mU/L). Ferritin was slightly elevated at 335 U/L (normal ≤ 300 μg/L), most probably in the context of inflammation as well as the chronic alcohol consumption. Urine analysis was normal. Human immunodeficiency virus (HIV) and viral hepatitis B and C serology as well as ELISPOT for latent tuberculosis were negative.

### Imaging

**Fig. 2:**
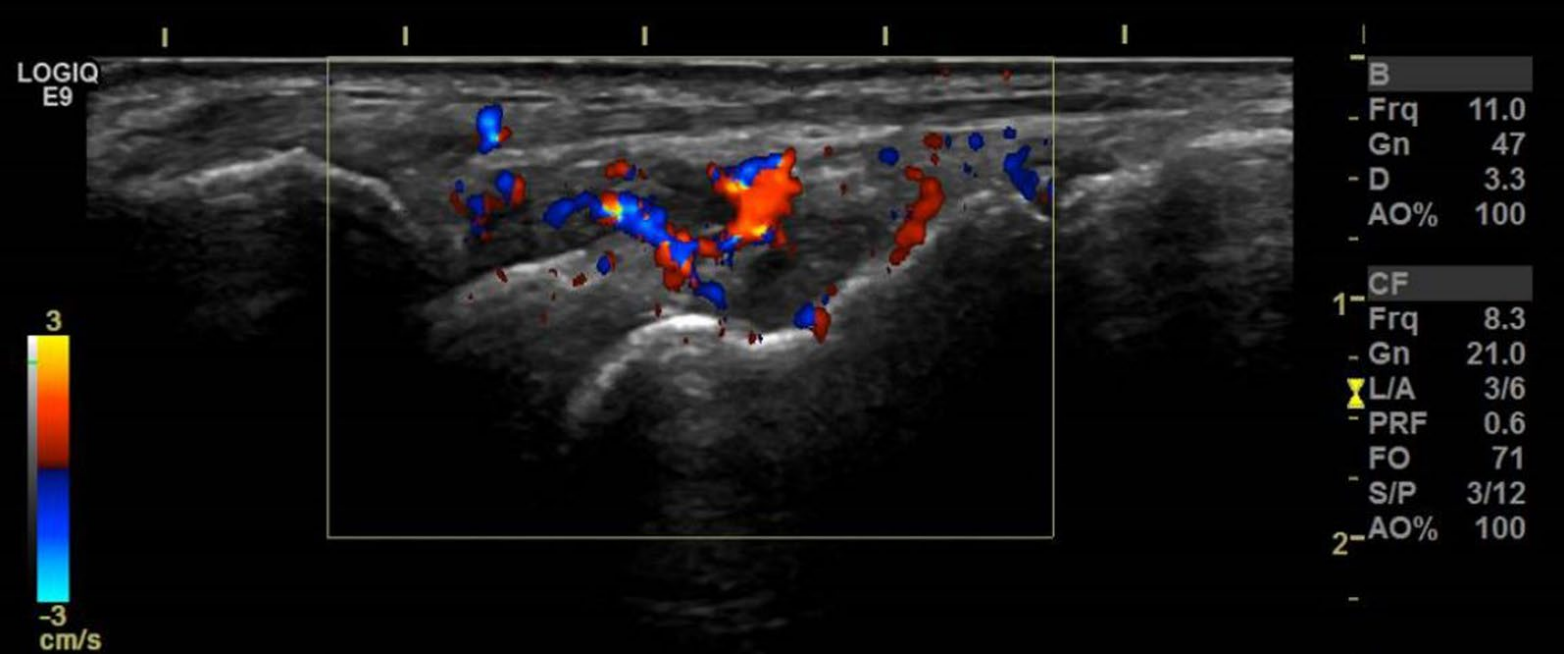
Ultrasound of the right wrist, dorsomedial aspect, with high color Doppler activity

Hand X-rays revealed no erosion or chondrocalcinosis, and chest X-rays showed no sign of sarcoidosis or latent tuberculosis. Magnetic resonance imaging (MRI) and X-rays showed no signs of spondylarthritis. Besides the flexor tenosynovitis, ultrasound examination (Fig. 2) revealed a synovitis of the wrist and of metacarpophalangeal joints (MCP) 2–4 of the right hand, a small, asymptomatic effusion of the right knee, and a bilateral subacromial bursitis.

**Fig. 3:**
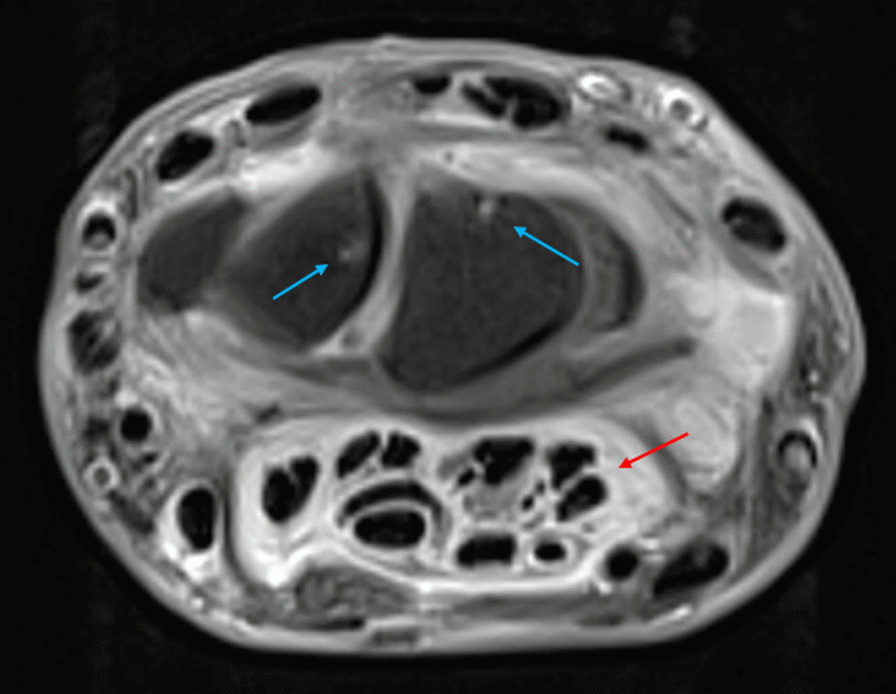
Magnetic resonance imaging of the right hand, transversal view: red arrow shows tenosynovitis of superficial and deep flexor tendons; blue arrows show carpal bones erosions

The hand magnetic resonance imaging (Fig. 3) confirmed the highly inflammatory finger flexor tenosynovitis and an revealed an erosive carpal synovitis.

### Microbiological and histological findings

Histopathological analysis of the first two synovectomy specimens showed a lymphocytic infiltrate with rare granulomatous reaction, with a negative periodic acid Schiff stain in the second synovectomy. No microbiological investigation was done on the first specimen; Gram stain and the standard bacteriological cultures, both aerobic and anaerobic, were negative in the second synovectomy. Further analysis of the synovial fluid of the wrist was negative for mycobacteria-specific culture, auramine stain, and crystals. In the absence of fever, blood cultures were not obtained.

A preliminary diagnosis of seronegative polyarthritis was made. As differential diagnoses, we discussed polymyalgia rheumatica (discarded because the massive tenosynovitis did not fit with the clinical picture) as well as peripheral spondylarthritis (less probable because the arthritis was mainly symmetrical, located on the upper limbs, and the HLA B-27 was negative).

### Treatment

**Fig. 4:**
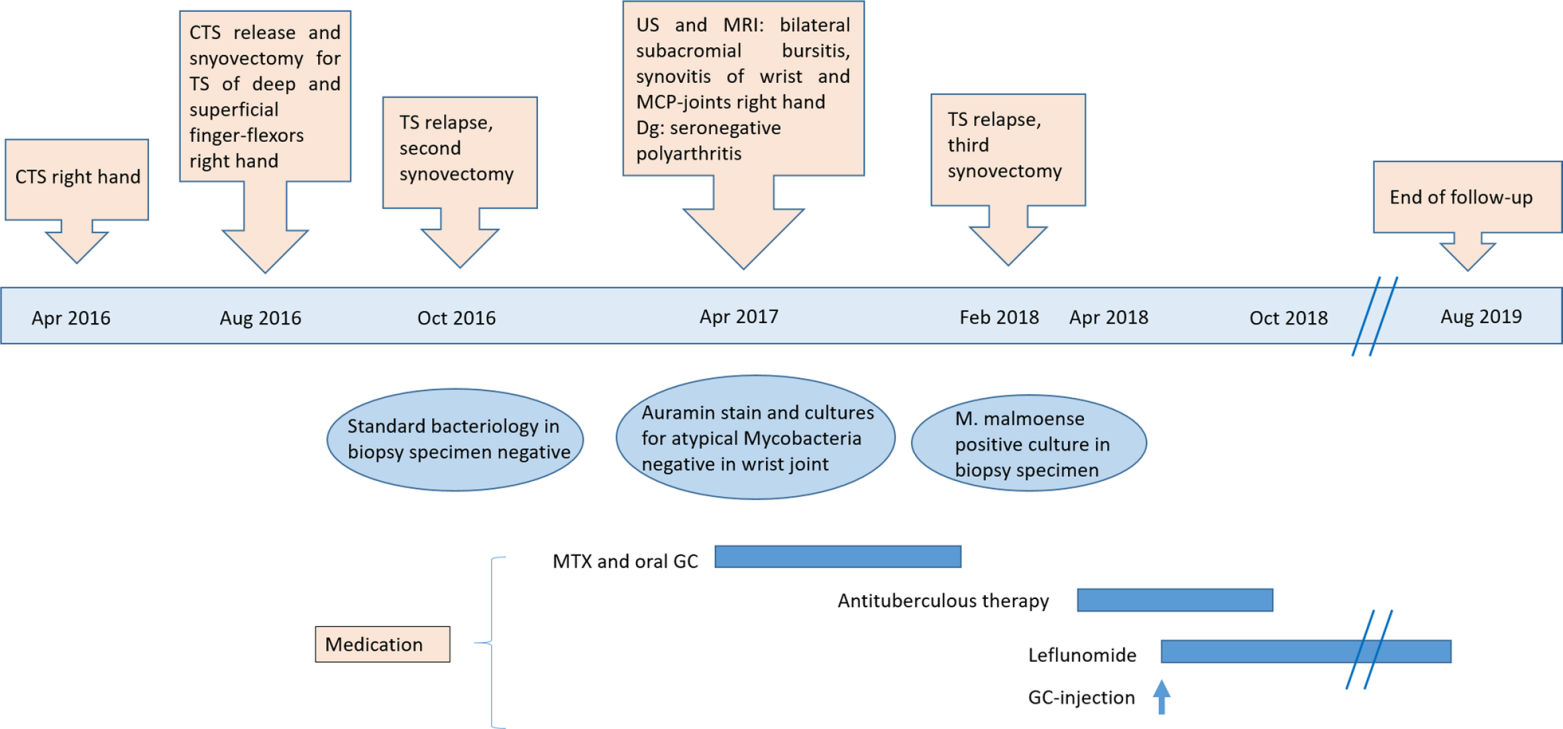
Timeline of diagnostic and treatment from first disease manifestation to the end of follow-up. *CTS* carpal tunnel syndrome, *TS* tenosynovitis, *US* ultrasound, *MRI* magnetic resonance imaging, *MCP* metacarpophalangeal, *MTX* methotrexate, *GC* glucocorticoids

Glucocorticoids (prednisolone up to 30 mg/day) and methotrexate (15 mg subcutaneously weekly) were prescribed, resulting in no significant clinical improvement of the wrist, but leading to remission in the other joints and normalization of inflammatory parameters. The treatment was stopped 10 months later because of side effects. Due to a massive worsening of the tenosynovitis, a third synovectomy was performed during the same period, showing nontuberculous granulomatous inflammatory infiltrates. The analysis was negative for mycobacteria, including auramine stain, *Mycobacterium tuberculosis* complex real-time polymerase chain reaction (PCR) and pan-mycobacterial PCR (for the detection of NTM). *Mycobacterium malmoense* was identified after 26 days of incubation with the mycobacteria-specific culture, with antimicrobial susceptibility to clarithromycin, ethambutol, moxifloxacin, linezolid, rifabutin, clofazimine, and rifampicin. We therefore concluded that the cause of the tenosynovitis was infectious, and initiated an antituberculous therapy with ethambutol (600 mg/day PO), rifampicin (450 mg/day PO), and clarithromycin (500 mg twice daily PO). Clarithromycin was replaced by azithromycin (350 mg/day PO) soon afterwards because of a severe glossitis. Two months under treatment, in the absence of any improvement, a concomitant rheumatic inflammatory component was suspected, and after careful consideration and in agreement with the infectious disease specialists, low-dose leflunomide (10 mg/day PO) was added. We equally performed a glucocorticoid injection with betamethasone of the flexor tendon sheath to avoid a fourth synovectomy, and the swelling partially improved after a further month. After 6 months of antituberculous treatment, the patient regained nearly normal wrist mobility. Although some degree of tenosynovitis persisted, the patient decided to stop antimicrobial therapy against medical advice, but agreed to increase the leflunomide dose to 20 mg/day. At the last visit, 10 months after the antituberculous therapy and after 1 year on leflunomide, the situation remained stable. Despite our recommendation, the patient interrupted the treatment, as well as the follow-up. Contacted by phone 2 years later, she reported an unchanged situation, with good functional status of her right hand and absence of joint pain elsewhere. A summary of the clinical case is depicted in Fig. 4.

## Discussion and conclusion

We present an extremely rare clinical case of *M. malmoense* tenosynovitis, occurring in parallel with polyarthritis. Unlike the two other rheumatologic patients suffering from *M. malmoense* tenosynovitis described in the literature [5, 7], our patient had no immunosuppressive treatment. The rapid relapse of tenosynovitis after the first and second tenosynovectomy is also unusual, as this bacterium is a slow-growing NTM species. Equally, the patient did not present any general symptoms, such as fever, fatigue, or weight loss. These circumstances led to a long diagnostic delay of about 2 years, yet with a satisfactory response to antituberculous therapy.

Ten NTM species can cause osteoarticular symptoms, especially tenosynovitis of the hand. Although *M. marinum* and *M. kansasii* are by far the most frequently identified, other species such as *M. malmoense* can also lead to tenosynovitis. The contamination results either from direct trauma, surgery, glucocorticoid injection, or occult inoculation from an environmental source [8].

To our knowledge, there are only 11 cases of *M. malmoense* tenosynovitis described in the literature [5]. Five of these patients were immunocompromised. Only two of the total number of patients had a previous rheumatic disease—rheumatoid arthritis [5] and chronic relapsing polychondritis [7], respectively, and they were both under glucocorticoid therapy. The three other immunocompromised patients suffered from asthma (one patient) or had kidney transplantation (two patients). The source of contamination was identified in only 3 out of the 11 patients and consisted of a corticosteroid injection in two patients, and a trauma in the third one, which is in line with the literature. Interestingly, none of these three patients was under immunosuppression.

As for our patient, contamination with *M. malmoense* during the first surgery could be practically ruled out, as the tenosynovitis had already been present at that time. Our patient was possibly contaminated with the bacteria through the contact with her cacti. In regard to the microbiological investigations, it is important to note that a negative auramine stain does not exclude an infection [9], which was precisely the case in our patient. Indeed, the sensitivity of acid-fast bacilli detection is very low in this type of specimen. The pan-mycobacterial PCR targeting the 16S rRNA can shorten the time to diagnose NTM infections when compared with culture. In the present case, the pan-mycobacterial PCR was negative. Interestingly, the sensitivity of this PCR is limited in smear-negative specimens [3]. Furthermore, being a slow-growing NTM, *M. malmoense* requires 8–12 weeks incubation time under specific conditions before growth is detected. Routine microbiological analysis cannot identify a NTM, so strong clinical suspicion is required to prompt the diagnosis.

One of the difficulties of our case consisted in the concomitant presence of polyarthritis and infectious tenosynovitis. As we tried to fit the clinical picture under the same entity, the polyarthritis, the final diagnosis and the correct treatment for the tenosynovitis were delayed by many months. We therefore estimate that our case illustrates well the problems one might encounter when facing an unusual association of diseases, in terms of diagnosis as well as treatment. Equally, our case stresses the importance of having a high clinical suspicion of infectious tenosynovitis, especially with nontuberculous mycobacteria, even in the absence of immunosuppression. In addition, several samples might be necessary to make the diagnosis. In our case, microbiological analyses were repeatedly negative and the bacterium was only identified in the third biopsy specimen.

A timely diagnosis helps prevent the morbidity and potential disability resulting from unnecessary and ineffective interventions. The treatment of *M. malmoense* tenosynovitis is not well established, but it seems that antituberculous therapy combined with surgical debridement is the most adequate approach [8]. Most authors believe that combination of several drugs, including ethambutol and rifampicin, has the highest success rate. Clarithromycin is also effective, reaches excellent tissue concentration, and may be given additionally. This was the regimen used in our patient. While treatment guidelines are well standardized for pulmonary NTM infection, the optimal duration of antituberculous therapy for tenosynovitis is not known. A minimal duration of 6–9 months seems appropriate, but longer periods of 12 months or more are warranted in difficult cases [10, 11].

The presence of concomitant arthritis complicates the treatment, as in our case, because one has to put into balance the need for immunosuppression and the need to control the infection, as the immunosuppressive therapy may influence the efficacy of antituberculous agents. Treating the infection has priority over the treatment of the inflammatory condition [8]. One should strongly consider stopping immunosuppressive therapy, especially tumor necrosis factor (TNF) inhibitors, or choosing less potent medication.

A limitation of our case is the diagnostic doubt about the atypical concomitant rheumatic disease, the clinical presentation being one of nonsymmetrical seronegative polyarthritis associated with bilateral shoulder bursitis. Nevertheless, the excellent clinical improvement of the joints (other than the right hand) under immunosuppression validates the diagnosis of a genuine arthritis occurring simultaneously with the infection.

Another limitation is the relatively short observation period of 1 year and a half, as the patient decided to stop the treatment prematurely and interrupted the follow-up.

In conclusion, this case highlights the importance of having a high clinical suspicion of an atypical infection in patients with inflammatory tenosynovitis not responding to usual care. Repeated microbiological analysis is sometimes necessary [8], as NMT are difficult to isolate and grow. Given the increasing occurrence of the slow-growing NTM tenosynovitis, in case of an unclear rheumatic condition, it is important to rule out this type of infection before initiating any immunosuppressive therapy.

## Data Availability

Data are available on demand.

## Abbreviations

NTM: Nontuberculous mycobacteria
MRI: Magnetic resonance imaging
